# Diosgenin, a Steroidal Saponin, Inhibits Migration and Invasion of Human Prostate Cancer PC-3 Cells by Reducing Matrix Metalloproteinases Expression

**DOI:** 10.1371/journal.pone.0020164

**Published:** 2011-05-23

**Authors:** Pin-Shern Chen, Yuan-Wei Shih, Hsiang-Ching Huang, Hsing-Wen Cheng

**Affiliations:** 1 Department of Biotechnology, Chia Nan University of Pharmacy and Science, Tainan, Taiwan; 2 Department of Biological Science and Technology, Chung Hwa University of Medical Technology, Tainan, Taiwan; 3 Graduate Institute of Biomedical Science, Chung Hwa University of Medical Technology, Tainan, Taiwan; Yale Medical School, United States of America

## Abstract

**Background:**

Diosgenin, a steroidal saponin obtained from fenugreek (*Trigonella foenum graecum*), was found to exert anti-carcinogenic properties, such as inhibiting proliferation and inducing apoptosis in a variety of tumor cells. However, the effect of diosgenin on cancer metastasis remains unclear. The aim of the study is to examine the effect of diosgenin on migration and invasion in human prostate cancer PC-3 cells.

**Methods and Principal Findings:**

Diosgenin inhibited proliferation of PC-3 cells in a dose-dependent manner. When treated with non-toxic doses of diosgenin, cell migration and invasion were markedly suppressed by in vitro wound healing assay and Boyden chamber invasion assay, respectively. Furthermore, diosgenin reduced the activities of matrix metalloproteinase-2 (MMP-2) and MMP-9 by gelatin zymography assay. The mRNA level of MMP-2, -9, -7 and extracellular inducer of matrix metalloproteinase (EMMPRIN) were also suppressed while tissue inhibitor of metalloproteinase-2 (TIMP-2) was increased by diosgenin. In addition, diosgenin abolished the expression of vascular endothelial growth factor (VEGF) in PC-3 cells and tube formation of endothelial cells. Our immunoblotting assays indicated that diosgenin potently suppressed the phosphorylation of phosphatidylinositide-3 kinase (PI3K), Akt, extracellular signal regulating kinase (ERK) and c-Jun N-terminal kinase (JNK). In addition, diosgenin significantly decreased the nuclear level of nuclear factor kappa B (NF-*κ*B), suggesting that diosgenin inhibited NF-κB activity.

**Conclusion/Significance:**

The results suggested that diosgenin inhibited migration and invasion of PC-3 cells by reducing MMPs expression. It also inhibited ERK, JNK and PI3K/Akt signaling pathways as well as NF-*κ*B activity. These findings reveal new therapeutic potential for diosgenin in anti-metastatic therapy.

## Introduction

Diosgenin is a naturally occurring steroidal saponin present in a variety of plants including fenugreek (*Trigonella foenum graecum*) and roots of wild yam (*Dioscorea villosa*) [1]. Diosgenin has been used in traditional medicine as an antihypercholesterolemia, antihypertriacylglycerolemia, antidiabetes and antihyperglycemia agent [1], [2], [3], [4]. Several reports have showed that diosgenin inhibits proliferation and induces apoptosis in a wide variety of tumor cells of human colon [5], osteosarcoma [6], leukemia [7], erythroleukemia [8], breast [9], and liver [10]. The anti-cancer effect of diosgenin has been demonstrated through cell cycle arrest [7], [11], activation of p53 and caspase-3 [5], [7], [8], [12]. In addition, diosgenin inhibits NF-κB activity and NF-κB-regulated gene expression and subsequently reducing proliferation, invasion and osteoclastogenesis [6], [13]. Diosgenin also abolishes cyclooxygenase-2 [11] and lipoxygenase [14], which are implicated in carcinogenesis and as important targets for cancer chemoprevention and therapy. Therefore, diosgenin may possess the cancer chemotherapeutic potential and its activity involves multiple cellular and molecular targets.

Prostate cancer is one of the most commonly diagnosed tumors in men and the second leading cause of cancer mortality in the United States [15]. Although prostate cancer at the early stage can be treated with surgery and androgen-deprivation therapy, it eventually progress to more malignant, metastasis, and hormone refractory prostate cancer (HRPC), for which there is no curative therapy [16], [17]. Thus, development of innovative therapies for the treatment of prostate cancer is needed. Because advanced prostate cancer cell with highly invasive potential result in high morbidity and mortality rates, inhibition of invasion and metastasis might be a good approach for treatment of HRPC.

Cancer metastasis is a highly coordinated step-wise process that includes detachment of cells from the primary tumor, local proteolysis of the extracellular matrix (ECM), penetration through the basement membrane of capillary and lymphatic vessels, intravasation, and then invasion into new tissue and growth [18], [19]. The process of metastasis is promoted by expressing and secreting various proteolytic enzymes that can degrade most ECM components. Matrix metalloproteinases (MMPs), a family of Zn-dependent endopeptidases, are the major proteases participating in tumor cell migration, spreading, tissue invasion and metastasis [20]. Among the MMPs, MMP-2 and MMP-9 are key enzymes for degrading type IV collagen and contribute to the process of metastasis [21], [22]. MMP-2 and MMP-9 are also capable of cleaving type I collagen [23], [24], the major component forming a lattice structure in stroma [25]. The activation of these enzymes have been associated with increasing tumor metastasis, suggesting an central functional role for these proteases in the metastatic process [26]. Proteolytic degradation of stromal microenvironment plays a critical role in promoting invasion. To acquire detailed information on cancer cell invasion on the stroma, type I collagen is used in Boyden chamber invasion assay in the present study.

In addition, proteolytic degradation of ECM in tumor metastasis can be regulated by other proteins such as extracellular inducer of matrix metalloproteinase (EMMPRIN) and tissue inhibitor of metalloproteinases (TIMPs). EMMPRIN is a multifunctional glycoprotein that can modify the tumor microenvironment by activating proteinases, inducing angiogenic factors in tumor and stromal cells. EMMPRIN is able to regulate MMPs and be involved in the invasion and metastasis processes of prostate cancer cells [27]. The activities of most MMPs are regulated by TIMPs. The balance between MMP and TIMP levels is an important determinant of the net proteolytic activity [28].

Mitogen-activated protein kinase (MAPK) pathway has been known to participate in numerous signaling cascades that play important regulatory roles in cell growth, apoptosis, differentiation, and metastasis [29]. The diverse MAPK members are activated in response to various extracellular stimuli and have distinct downstream targets, thus stimulating cell migration, proteinase-induction, and angiogenesis, events that are essential for metastasis [30]. Extracellular signal regulating kinase (ERK1/2) and c-Jun N-terminal kinase (JNK), two major mammalian MAP kinases, have been implicated in cell migration and proteinase-induction, events that are essential for metastasis [30]. ERK1/2 and JNK play a central role in regulating the expression of MMPs [20]. Inhibition of the MAPK pathway might have the potential to prevent angiogenesis, proliferation, invasion, and metastasis for a wide range of tumors [31], [32], [33]. Metastasis is also regulated by the PI3K/Akt signaling pathway, which is involved in many cellular processes including cell survival, cell adhesion and metastasis [34], [35]. Inhibition of the MAPK and PI3K/Akt pathways may have the potential to prevent cancer cell proliferation, invasion, and metastasis [31], [33]. In addition, PI3K/Akt and MAPK signaling pathways play a central role in regulating the expression of MMPs by transcriptional factors, including NF-κB [34], [36], [37]. NF-κB is kept in an inactive form in the cytoplasm by inhibitory proteins called inhibitors of κB (IκB). In response to an activation signal, NF-κB is released from IκBα and translocates from the cytoplasm to the nucleus, where it binds to cognate sequences in the promoter region of a number of target genes and subsequently facilitates cell proliferation, angiogenesis, and metastasis. Thus, blocking PI3K/Akt and MAPK pathways as well as NF-κB provide potential targets for tumor therapeutic strategies.

Although diosgenin is implicated as a novel multitarget-based chemopreventive agent against several cancer cells, the role of diosgenin against tumor metastasis and angiogenesis is still unclear. The objective of this work is to examine the inhibitory effects and molecular mechanisms of diosgenin on metastasis. Since human prostate cancer PC-3 cell exhibits highly invasive and metastatic activity and has been used for investigating the biochemical changes in advanced prostatic cancer cells and in assessing their response to chemotherapeutic agents [38], PC-3 cell was used for the present experiments.

## Materials and Methods

### Reagents and Cell Culture

Diosgenin, dimethyl sulfoxide (DMSO), Tris-HCl, EDTA, SDS, phenylmethylsulfonyl fluoride (PMSF), Nonidet P-40, deoxycholic acid and sodium orthovanadate, were purchased from Sigma-Aldrich (St. Louis, MO). Protein assay kit was obtained from Bio-Rad Labs (Hercules, CA). Powdered Dulbecco's modified Eagle's medium (DMEM) was purchased from Gibco/BRL (Gaithersburg, MD). Total RNA extraction kit and PCR kit were from Viogene (Sunnyvale, CA). Antibodies against ERK, JNK, Akt, NF-κB (p65), C23 and phosphorylated proteins were purchased from Santa Cruz Biotechnology (Santa Cruz, CA). Antibodies against PI3K and phosphorylated PI3K were purchased from Cell Signaling Technology (Danvers, MA). Human prostate cancer cell lines PC-3 and was obtained from BCRC (Food Industry Research and Development Institute, Taiwan). Cells were maintained in DMEM supplemented with 10% fetal calf serum, 100 U/ml of penicillin and 100 µg/ml streptomycin, and incubated in a 5% CO_2_ humidified incubator at 37°C. Human umbilical vein endothelial cell (HUVEC), kindly provided from Dr. Hua-Lin Wu [39], was isolated from human umbilical cord veins as previously described [39], and cultured on 0.1% gelatin-coated dishes and maintained in M199 medium supplemented with 16% FBS, endothelial cell growth supplement and heparin sulfate (Upstate Biotechnology). HUVECs between passages 2 and 6 were used in all experiments. For diosgenin treatment, diosgenin was dissolved in ethanol and diluted with culture medium (the final concentration of ethanol was less than 0.2%).

### Cell Viability Assay

The assay was performed as described previously [40]. Briefly, cells were seeded in a 96-well plate and treated with diosgenin in triplicate. After 24 and 48 hrs of incubation, the medium was replaced with fresh medium containing 0.5 mg/ml MTT [3-(4,5-dimethylthiazol-2-yl)-2,5-diphenyltetrazolium bromide]. After 4 hrs, the supernatants were removed and the resulting MTT formazan was solubilized in DMSO and measured spectrophotometrically at 570 nm.

### Wound Healing Migration Assay

The assay was performed as described previously [41]. PC-3 cells were plated in a 12-well plate and grew to confluence. The monolayer culture was then scrape-wounded with a sterile micropipette tip to create a denuded zone (gap) of constant width. After removing the cellular debris with PBS, cells were exposed to various concentrations of diosgenin after 24 hrs. PC-3 cells migrated to the wounded region were observed by Olympus CK-2 inverted microscope and photographed (100× magnification). The wound area was measured by the program Image J (http://rsb.info.nih.gov/ij/). The percentage of wound closure was estimated by the following equation: Wound closure %  =  [1-(wound area at T_t_/wound area at T_0_) ×100%, where T_t_ is the time after wounding and T_0_ is the time immediately after wounding.

### Boyden Chamber Invasion Assay

Boyden chamber invasion assay was carried out as previously [41]. Briefly, the polycarbonate filter (8 µm pore) was pre-coated with type-I collagen (10 µg/ml). After treated with diosgenin for 24 hrs, cells (1×10^4^ cells/well) were added to the upper chamber in serum-free medium. The complete medium (containing 10% FBS) was applied to the lower chamber as chemoattractant. The chamber was incubated for 6 hrs at 37 °C. At the end of incubation, the cells in the upper surface of the membrane were carefully removed with a cotton swab and cells that invaded to the lower surface of the membrane were fixed with methanol and stained with 5% Giemsa solution. The invaded cells on the lower surface of the membrane filter were scored from five random fields under microscopy (200× magnification).

### Tube Formation Determination

The tube formation assay was performed as described. Briefly, a 15-well μ-Slides (ibidi, Germany) was coated with 10 µl of Matrigel which was allowed to solidify at 37 °C. To evaluate the effect of diosgenin, PC-3 cells were treated with various concentrations of diosgenin for 24 hrs and the conditioned medium were collected and subjected to tube formation assay. HUVEC were seeded on the Matrigel and cultured in conditioned medium of PC-3 cell for 6 hrs. The enclosed networks of complete tubes were counted and photographed under an inverted microscope. The tubular lengths of the cells were measured using the program Image J.

### RNA Extraction and Reverse Transcription PCR and Quantitative Real-Time PCR

Total RNA was extraction using total RNA extraction kit according to the manufacturer's instructions. Total RNA (1 µg) from each sample was subject to reverse transcription with oligo(dT) primers by PCR kit according to manufacturer's instruction. The synthesized cDNA was used for PCR amplification with the following primers [41]: MMP-9, 5′-gcacgacgtcttccagtacc-3′ (For), 5′-tcaactcactccgggaactc-3′ (Rev); MMP-2, 5′-ggactatgaccgggataagaaatatg-3′ (For), 5′-gggcaccttctgaatttcca-3′ (Rev); β-actin: 5′-TGTTACCAACTGGGACGACA-3′ (For), 5′-GGGGTGTTGAAGGTCTCAAA-3′ (Rev). cDNAs were amplified for 35 cycles and each PCR reaction condition was as follows: preparation step at 94°C for 5 min, denaturing step at 94°C for 30 sec, annealing step at 56°C for 30 sec, and polymerization step at 72°C for 30 sec. PCR products were analyzed by agarose gel electrophoresis. The mRNA expressions of MMP-2, -9, -7, EMMPRIN and TIMP-1,-2 were determined by quantitative real-time PCR which is conducted in StepOne system (Applied Biosystem, Foster City, CA, USA). Briefly, each amplification mixture (50 µl) contains 10 ng cDNA and 25 µl SYBR Green PCR Master Mix (Applied Biosystems). PCR conditions were as follows: 95 °C for 2 min, 40 cycles at 95 °C for 15 s and 60 °C for 45 s. The primer sequences for β-actin, MMP-2, MMP-9, MMP-7, EMMPRIN, TIMP-1, TIMP-2 and vascular endothelial growth factor (VEGF) were deduced from PrimerBank and listed in Table 1. PCR results were derived using the comparative C_T_ method.

**Table 1:** Primer pairs used in Quantitative Real-Time PCR.

Gene	Sequence (5′-3′)	Amplicon (bp)
MMP-2-F	CTTCCAAGTCTGGAGCGATGT	119
MMP-2-R	TACCGTCAAAGGGGTATCCAT	
MMP-9-F	GGGACGCAGACATCGTCATC	139
MMP-9-R	TCGTCATCGTCGAAATGGGC	
MMP-7-F	GGAGGAGATGCTCACTTCGAT	118
MMP-7-R	AGGAATGTCCCATACCCAAAGA	
EMMPRIN-F	CTACACATTGAGAACCTGAACAT	170
EMMPRIN-R	TTCTCGTAGATGAAGATGATGGT	
TIMP-1-F	CTTCTGCAATTCCGACCTCGT	127
TIMP-1-R	CCCTAAGGCTTGGAACCCTTT	
TIMP-2-F	AAGCGGTCAGTGAGAAGGAAG	153
TIMP-2-R	CACACACTACCGAGGAGGG	
β-actin-F	CATGTACGTTGCTATCCAGGC	250
β-actin-R	CTCCTTAATGTCACGCACGAT	

### Analysis of MMP-2 and MMP-9 Activities by Gelatin Zymography

The activities of MMP-2 and MMP-9 were assayed by gelatin zymography as described previously [41]. Briefly, subconfluent PC-3 cells were incubated with serum-free medium with various concentrations of diosgenin for 24 hrs. The conditioned medium was then harvested and concentrated by ultra-filtration centrifugation. The sample (20 µg) was mixed with loading buffer and subjected to 10% SDS-polyacrylamide gel containing 0.1% gelatin. Electrophoresis was performed at 100 V for 3 h at 4°C. Gels were then washed with washing buffer (2.5% Triton X-100 in dd H_2_O) at room temperature to remove SDS, followed by incubation at 37°C in reaction buffer (40 mM Tris-HCl, pH 8.0, 10 mM CaCl_2_, 0.02% NaN_3_). After 16 h, the gels were stained with Comassie blue R-250 (0.125% Comassie blue R-250, 50% methanol, 10% acetic acid) for 1 h and destained with destaining solution (20% methanol, 10% acetic acid, 70% ddH_2_O) until the clear bands were visualized.

### Nuclear Protein Extraction

The nuclear proteins were prepared as previously described [41]. Briefly, cells were washed with ice-cold PBS, centrifuged, and resuspended in hypotonic buffer (10 mM HEPES, pH 7.9, 1.5 mM MgCl_2_, 10 mM KCl, 0.05% NP-40, 0.5 mM DTT and 0.5 mM PMSF). The nuclei were centrifuged for 10 min at 3000 rpm at 4°C. The pellet was then resuspended in nuclear extract buffer (20 mM HEPES, pH 7.9, 1.5 mM MgCl_2_, 420 mM NaCl, 0.2 mM EDTA, 0.5 mM DTT, 0.5 mM PMSF and 25% glycerol) and incubated for 30 min on ice. After another centrifugation at 14,000 rpm for 10 min, the supernatant containing the nuclear protein was transferred into a prechilled microcentrifuge tube. The extracts were stored at –80°C.

### Western Blot

After being treated with diosgenin, PC-3 cells were washed twice with PBS and treated with extraction buffer (50 mM Tris-Cl, pH 7.5, 150 mM NaCl, 0.1% SDS, 1% NP-40, and 0.5% deoxycholic acid). The cell extractions were collected and centrifuged at 10,000 × g for 10 min at 4°C, and the supernatants were collected as cell lysates. The cell lysates were subjected to SDS-PAGE, and transferred to nitrocellulose membranes (Millipore, Bedford, MA). The membranes were blocked with 5% (w/v) non-fat milk in PBS containing 0.1% Tween-20, and then blotted with primary antibody. Subsequently, the membranes were incubated with an appropriate secondary antibody (horseradish peroxidase-conjugated goat anti-mouse or anti-rabbit IgG). The immuno-detected proteins were then revealed by enhanced chemiluminescence.

### Statistical Analysis

Data were expressed as mean ± standard deviation. Statistical significance was analyzed by one-way ANOVA. If the significance was observed, the Dunnett's post-hoc test was used to determine the difference between treatment groups and untreated group, with values of *p*<0.05 considered statistically significant.

## Results

### Cytotoxic Effect of Diosgenin in PC-3 Cells

**Figure 1:**
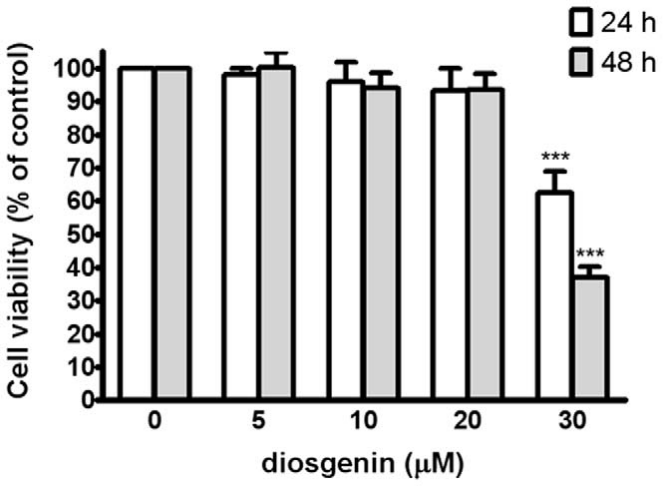
Effect of diosgenin on viabilities of PC-3 cell. Cells were treated with various concentrations of diosgenin for 24 h and 48 h. Cell viability is presented as mean ± S.D. of four independent experiments. ***p<0.001 compared with the untreated control.

We first elucidated the cytotoxic effect of diosgenin on prostate cancer cells PC-3 (Fig. 1). We demonstrated that treated of diosgenin at concentration below 20 µM for 24 or 48 hrs did not affect viability of PC-3 cell significantly. Viability of PC-3 cell was significantly decreased by diosgenin at 30 µM. The data indicated that treatment with diosgenin at doses of no more than 20 µM for 24 and 48 hrs did not cause cytotoxicity of PC-3 cells.

### Diosgenin Inhibits Migration in PC-3 Cells

**Figure 2:**
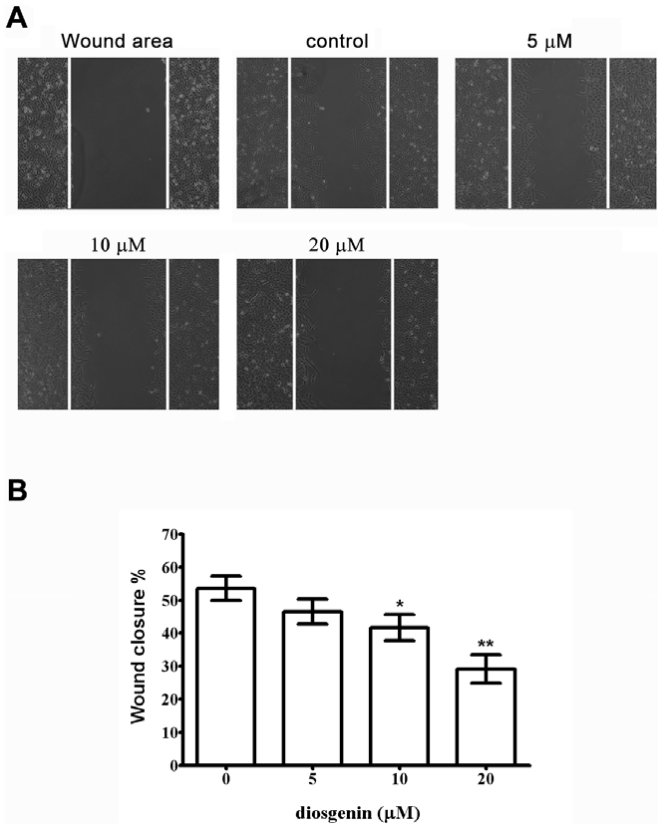
Effect of diosgenin on migration of PC-3 cells. Cell monolayers were scraped by a sterile micropipette tip and the cells were treated with various concentrations of diosgenin for 24 h. (A) Cells migrated to the wounded region were photographed (100× magnification). (B) The wound area of the cell cultures were quantified in four fields in each treatment, and data were calculated from three independent experiments. Data are presented as as mean ± S.D. of three independent experiments. *p<0.05, **p<0.01, compared with the untreated control.

Because a higher concentration of diosgenin was toxic, we investigate the inhibitory effect of diosgenin on migration and invasion of PC-3 cells using non-toxic doses. After incubation with different concentrations of diosgenin for 24 hrs, diosgenin suppressed migration of PC-3 cells to the denuded zone in a dose-dependent manner (Fig. 2, A and B). These results revealed that diosgenin inhibited the motility of PC-3 cells significantly.

### Diosgenin Inhibits Invasion in PC-3 cells

**Figure 3:**
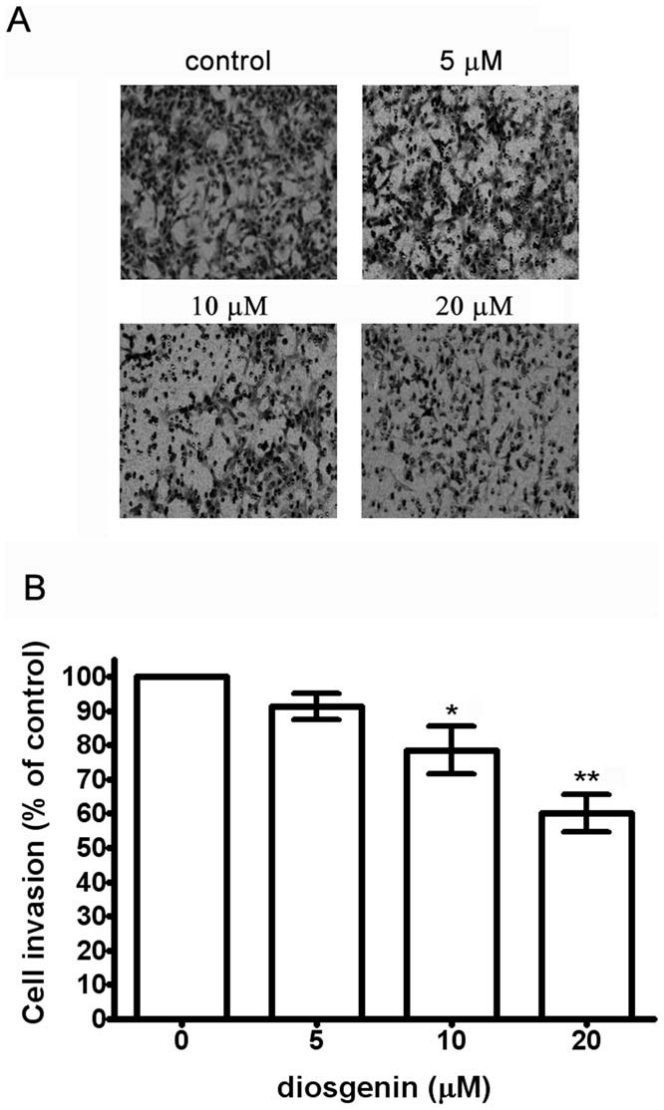
Effect of diosgenin on invasion of PC-3 cells. Cells were treated with various concentrations of diosgenin for 24 h and cell invasion assay was performed. (A) The invaded cells were photographed (200× magnification). (B) The invaded PC-3 cells were counted in five random fields in each treatment, and data were calculated from three independent experiments. Data are presented as mean ± S.D. of three independent experiments. *p<0.05, **p<0.01, compared with the untreated control.

To elucidate the inhibitory effect of diosgenin on the invasion of PC-3 cells across the extracellular matrix, the cells that invaded through the type-I collagen-coated polycarbonate filter in the Boyden chamber were analyzed. The results showed that diosgenin suppressed invasion of PC-3 cells across the type-I collagen-coated filter in a dose-dependent manner. Treatment with diosgenin of 10 and 20 µM inhibited 22% and 40% of cell invasion, respectively (Fig. 3, A and B). The results indicated that diosgenin markedly inhibited invasion of PC-3 cells.

### Diosgenin Inhibits Activation and Expression of MMP-2 and MMP-9 in PC-3 cells

**Figure 4:**
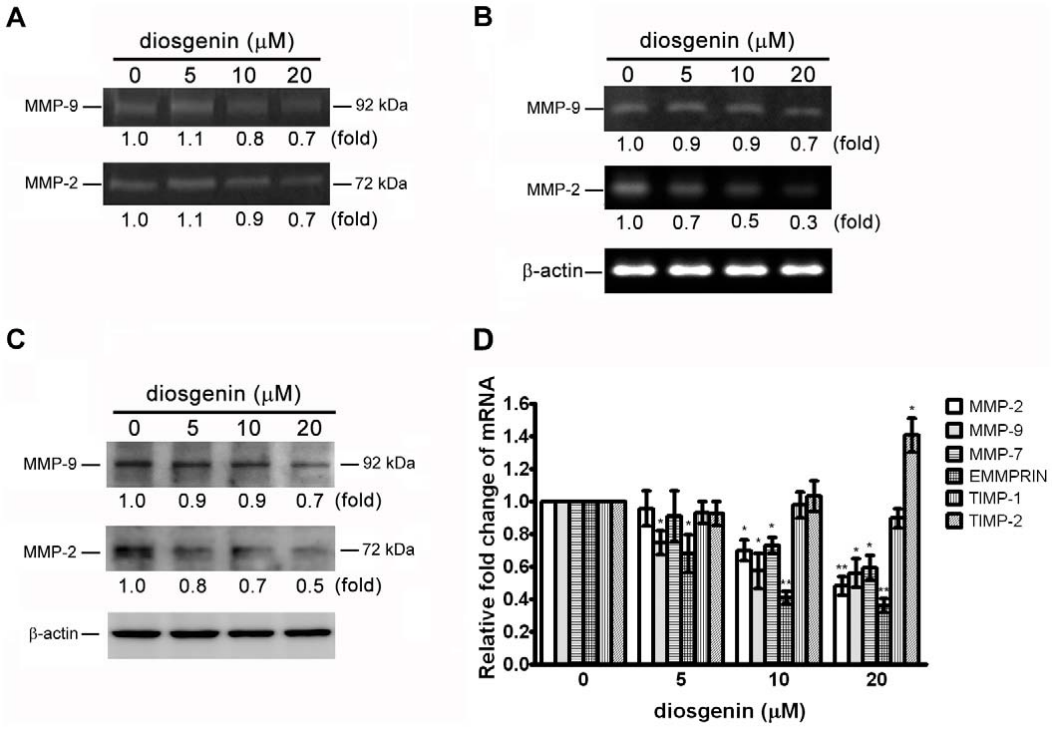
Effect of diosgenin on the activities and expressions of MMP-2/9/7, EMMPRIN and TIMP-1/2 in PC-3 cells. **(**A) PC-3 cells were treated with various concentrations of diosgenin for 24 h and the activities of MMP-9 and MMP-2 were determined by gelatin zymography. The expressions of MMP-9 and MMP-2 mRNA (B) and protein (C) were analyzed by RT-PCR and Western blotting, respectively. β-actin was used as an internal control. (D) The levels of MMP-2, -9, -7, EMMPRIN and TIMP-1,-2 mRNA were expressed as mean ± S.D. of three independent experiments. *p<0.05, **p<0.01, compared with the untreated control.

Since the activation of MMPs is crucial for ECM degradation, which is required for cell invasion, the effect of diosgenin on the activation of MMPs was investigated. After PC-3 cells were treated with various concentrations of diosgenin for 24 hrs in serum-free medium, the conditioned medium was collected, concentrated and assayed for MMP activity by gelatin zymography. The results showed that MMP-9 and MMP-2 activities were markedly reduced by 20 µM of diosgenin (Fig. 4A). We further demonstrated that diosgenin suppressed the expression of MMP-9 and MMP-2 mRNA and protein determined by RT-PCR and Western blotting, respectively (Fig. 4, B and C). The results indicated that both enzyme activities and expressions of MMP-9 and MMP-2 were inhibited by diosgenin.

### Diosgenin Inhibits mRNA Expression of MMP-2, MMP-9, MMP-7 and extracellular inducer of matrix metalloproteinase (EMMPRIN) while Induces TIMP-2 Expression

In order to elucidate the effect of diosgenin on expression of genes involved in ECM degradation, mRNA levels of MMP-2, MMP-9, MMP-7, EMMPRIN, TIMP-1 and TIMP-2 were analyzed by quantitative real-time PCR. The results demonstrated that diosgenin inhibited the mRNA expression of MMP-2, MMP-9, MMP-7 and EMMPRIN in a dose-dependent manner. Diosgenin also elevated the expression of TIMP-2, which is known to block the proteolytic potential of MMPs (Fig. 4D). The results suggested that diosgenin might affect the expression of genes involved in proteolytic activation.

### Diosgenin inhibited PC-3 cell induced Human Umbilical Vein Endothelial Cell (HUVEC) tube formation

**Figure 5:**
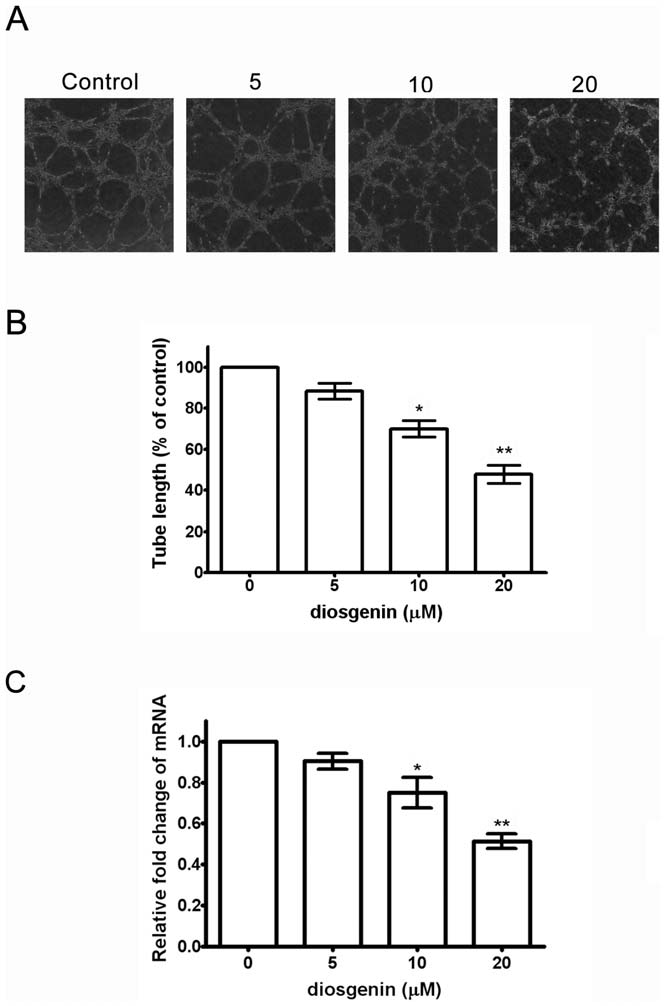
Effect of diosgenin on PC-3 induced tube formation of endothelial cell and VEGF expression. (A) HUVECs were seeded onto Matrigel and incubated with conditioned medium from PC-3 cells treated with diosgenin for 24 h. After 6 h, the enclosed networks of complete tubes were photographed (100× magnification). (B) The tubular lengths of the cells were measured using Image J software. (C) The mRNA expression of VEGF was determined and presented as mean ± S.D. of three independent experiments. *p<0.05, **p<0.01, compared with the untreated control.

Tube formation of endothelial cell is one of the crucial steps in angiogenesis associated with the cancer progression and metastasis. In order to examine the inhibitory effect of diosgenin on PC-3 cell induced angiogenesis, we performed in vitro tube formation of HUVEC by conditioned medium of PC-3 cells. HUVEC grown on Matrigel was treated with the conditioned media from PC-3 cells treated with diosgenin for 24 hrs, and tube formation were evaluated. The results demonstrated conditioned media of PC-3 cells induced tube formation of HUVEC, and conditioned media from cells exposed to diosgenin suppressed tube formation of HUVEC in a dose-dependent manner (Fig. 5, A and B). The results suggested that diosgenin might suppress PC-3 cell induced angiogenesis in vitro. Because PC-3 cells induce angiogenesis through expressing angiogenic factors such as VEGF, we evaluate whether diosgenin inhibits expression of VEGF in PC-3 cells. Results demonstrated that mRNA expression of VEGF in PC-3 cells was markedly decreased by diosgenin in a dose-dependent manner (Fig. 5C). Our data suggested that diosgenin inhibit PC-3 cell induced angiogenesis by suppressing VEGF expression.

### Diosgenin Inhibits phosphorylation of PI3K, Akt, ERK and JNK

**Figure 6:**
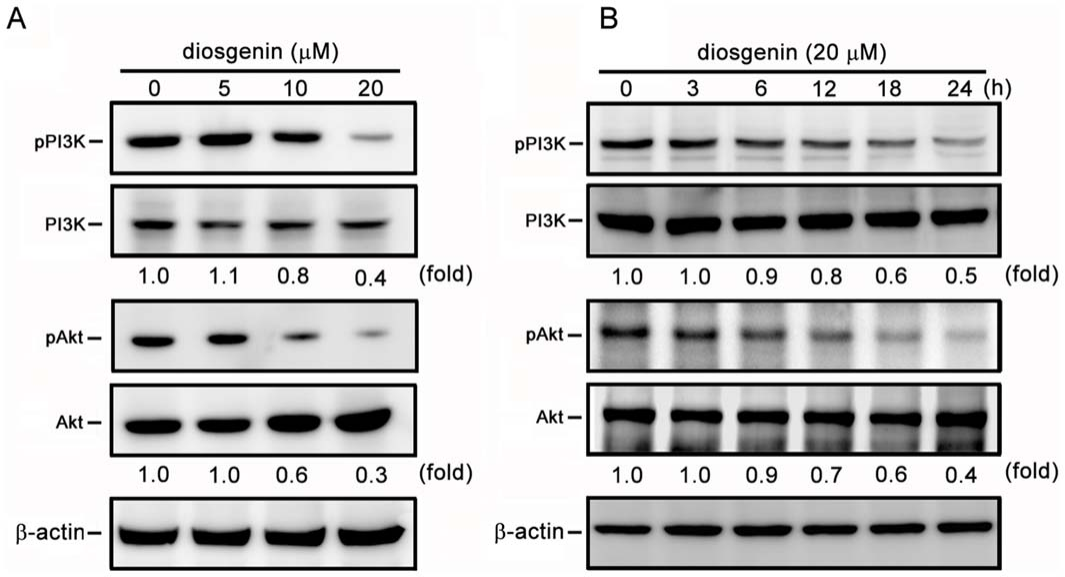
Effects of diosgenin on phosphorylation of PI3K and Akt. PC-3 cells were treated with various doses of diosgenin for 24 h (A), or 20 µM of diosgenin for 3, 6, 12, 18 and 24 h (B). The phosphorylation of PI3K and Akt was determined by SDS-PAGE and Western blotting. β-actin was used as a loading control.

**Figure 7:**
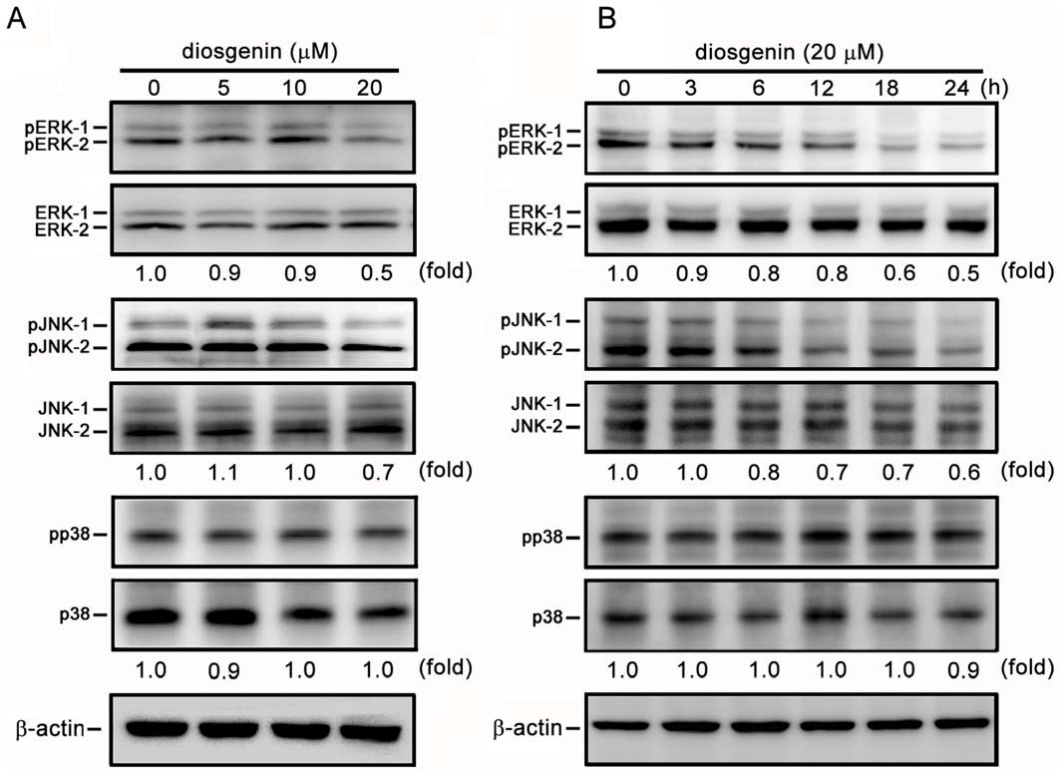
Effects of diosgenin on phosphorylation of ERK1/2, JNK1/2 and p38. PC-3 cells were treated with various doses of diosgenin for 24 h (A), or 20 µM of diosgenin for 3, 6, 12, 18 and 24 h (B). The phosphorylation of ERK1/2, JNK1/2 and p38 were determined by SDS-PAGE and Western blotting. β-actin was used as a loading control.

Several studies have indicated that the signaling proteins including PI3K, Akt and MAPK members are involved in the expression of MMPs and inducing metastasis [30], [34], [35]. The effects of diosgenin on the phosphorylated status of PI3K, Akt, ERK1/2, JNK1/2 and p38 in PC-3 cells were investigated. Data demonstrated that diosgenin reduced the phosphorylation of PI3K and Akt in a dose- and time-dependent manner (Fig. 6, A and B). In addition, diosgenin suppressed the phosphorylation of ERK1/2 and JNK1/2 in a dose- and time-dependent manner, while it did not alter the phosphorylation of p38 (Fig. 7, A and B).

**Figure 8:**
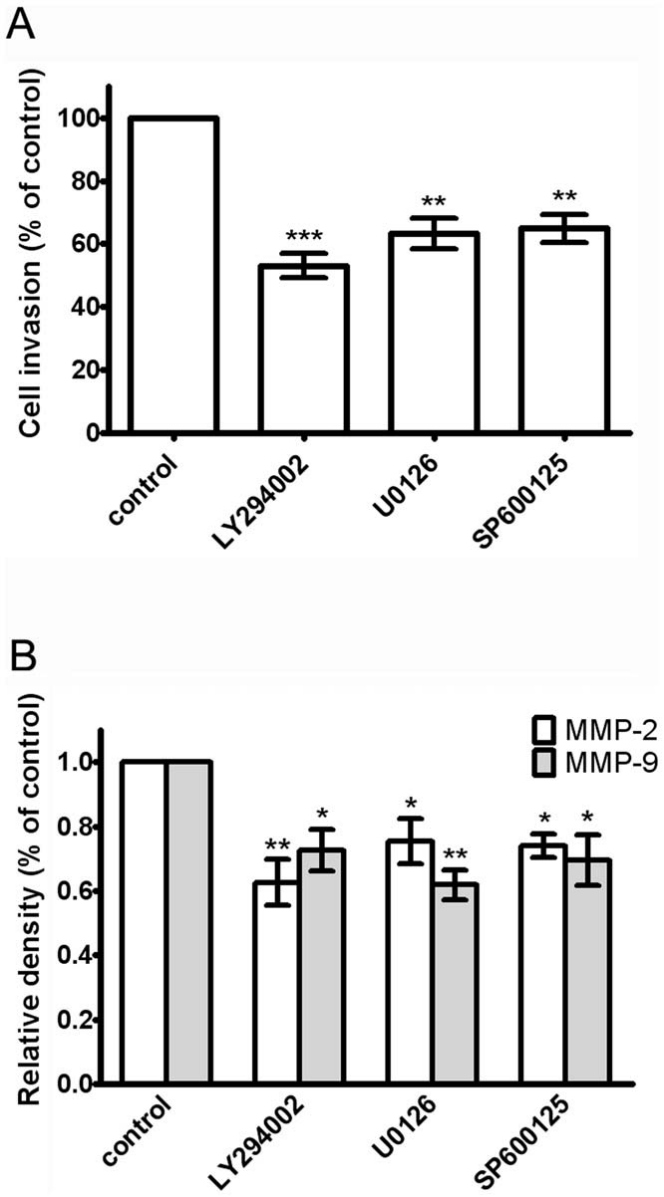
Effect of PI3K inhibitor (LY294002), ERK inhibitor (U0126) and JNK inhibitor (SP600125) on cell invasion and MMP-2/9 expression of PC-3 cells. (A) Cells were treated with LY294002, U0126 and SP600125 for 24 h and the cell invasive abilities were performed by Boyden chamber invasion assay. (B) The expression of MMP-2, -9 mRNA were determined and expressed as mean ± S.D. of three independent experiments. **p<0.01, ***p<0.001, compared with the untreated control.

To further investigate whether the inhibition of cell invasion and MMP-2/9 expression were through inhibition of the ERK1/2, JNK1/2 and PI3K signaling pathways, PC-3 cells were treated with a PI3K inhibitor (LY294002; 20 µM), ERK inhibitor (U0126; 20 µM) and JNK inhibitor (SP600125; 20 µM) for 24 hrs. Results showed that treatment of LY294002, U0126 and SP600125 reduced cell invasion and MMP-2/9 expression significantly (Fig. 8, A and B), suggesting that the inhibition of cell invasion and MMP-2/9 expression by diosgenin could partly occur through suppressing PI3K, ERK and JNK pathways.

### Diosgenin Downregulates the Nuclear Content of NF-κB in PC-3 Cells

**Figure 9:**
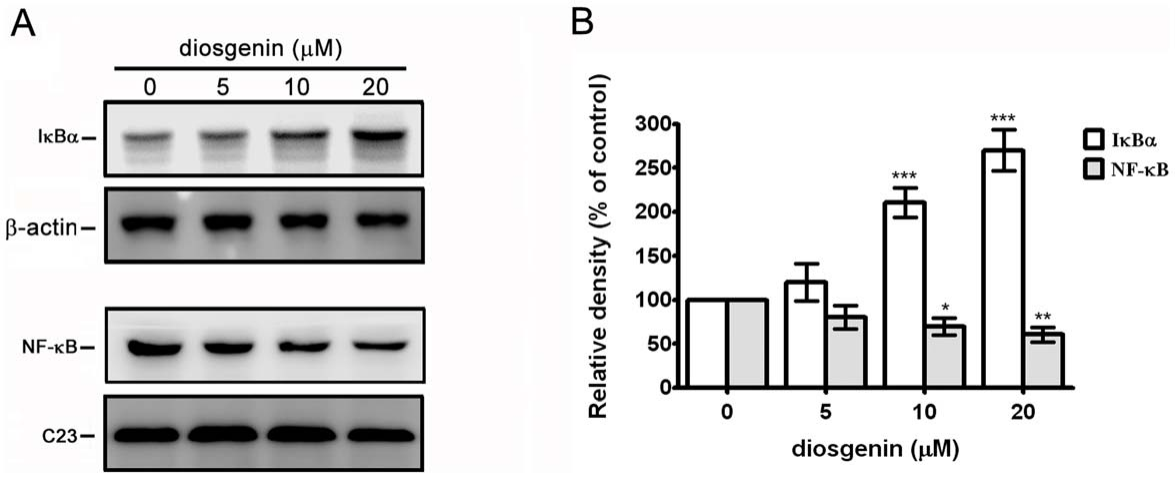
Effects of diosgenin on NF-κB activation. PC-3 cells were treated with various doses of diosgenin for 24 h. (A) The cytosolic and nuclear extracts were prepared and analyzed for IκBα degradation and NF-κB p65 translocation. β-actin and C23 were used as a cytosolic and nuclear protein loading control, respectively. (B) Determined levels of IκBα and NF-κB were quantified by densitometric analysis. The densitometric results were expressed as mean ± S.D. of three independent experiments. *p<0.05, **p<0.01, ***p<0.001, compared with the untreated control.

To investigate the inhibitory effect of diosgenin on the activity of NF-κB, the amount of IκBα in the cytosolic extracts and NF-κB in the cell nuclear extracts were measured by Western blotting. Data revealed that diosgenin-treated PC-3 cells demonstrated an increase in the cytosolic protein level of IκBα and a decrease in the nuclear protein level of NF-κB (Fig. 9). The results implicated that diosgenin significantly inhibited NF-κB activity.

## Discussion

Diosgenin has been shown to possess anti-carcinogenic potentials, such as inhibiting cell growth and inducing apoptosis of various cancer cell lines [5], [6], [7], [8], [9], [10]. In the present study, we provided evidences that diosgenin was able to inhibit metastasis *in vitro*, such as migration and invasion in human prostate cancer PC-3 cells, suggesting that diosgenin might possess anti-metastatic potential.

We demonstrated that diosgenin suppressed proliferation of PC-3 cells significantly at the concentration of 30 µM. When PC-3 cells were treated with diosgenin at non-toxic doses (below 20 µM), migration and invasion were inhibited. These results implied that the inhibitory effects of diosgenin on PC-3 cell migration and invasion were not due to its cytotoxic effect.

Cancer metastasis requires migration of cancer cells. During cell migration, pericellular proteolysis of ECM is important for cell protrusion. The proteolytic degradation of ECM mediated by extracellular proteases, such as MMPs, is required for prostate cancer cell migration and invasion. Among them, MMP-2, MMP-9 and MMP-7 play a critical role in prostate cancer progression. Expression of MMP-2 and MMP-9 are associated with prostate cancer progression [42], [43]. Inhibition of MMP-2 and MMP-9 expression suppress the metastatic potential of prostate cancer [32], [44]. MMP-7 possesses proteolytic activity against a variety of ECM substrates, including collagens, proteoglycans, elastin, laminin, fibronectin, and casein. MMP-7 is also produced by several malignant tumor cells including prostate, gastric, head and neck, lung, hepatocellular, and colorectal carcinomas [45], [46]. Overexpression MMP-7 promotes invasion of prostate cancer cells [47]. In addition, EMMPRIN is able to regulate MMPs and be involved in the invasion and metastasis processes of prostate cancer cells [27]. Inhibition of EMMPRIN expression reduces tumor cell invasion in human prostate cancer cell [48]. TIMPs, the regulator of MMPs, are also involved in tumor progression, invasion, metastasis and angiogenesis [49], [50]. In the present study, we showed that diosgenin inhibited migration and invasion of PC-3 cells. Treatment with diosgenin of 10 and 20 µM for 24 hrs diminished the activities and expression of MMP-2 and MMP-9 significantly. Diosgenin also inhibited mRNA expression of MMP-7 and EMMPRIN in PC-3 cells. Besides, the expression of TIMP-2 was increased by diosgenin. These results suggested that the inhibition of MMP-2/9/7- and EMMPRIN-mediated enzymatically degradative process and induction of TIMP-2 expression might be attributed to the anti-invasive effect of diosgenin. The inhibitory effect of diosgenin on MMPs may be, at least in part, responsible for its anti-metastatic potential.

Angiogenesis, a critical step in tumor growth and metastasis, is regulated by a variety of angiogenic factors, such as VEGF [51]. In the present study, our data demonstrated that diosgenin inhibited tube formation of endothelial cell, which is one of the first step in angiogenesis. We further indicated that this inhibitory effect of diosgenin could be mediated by the suppression of expression of VEGF from prostate PC-3 cells. In agreement with our observation, recent studies revealed that inhibition of tube formation of endothelial cell through the suppression of VEGF expression from a variety of cancer cells [44], [52], [53]. Therefore, we suggested that diosgenin inhibited VEGF expression within PC-3 cells and subsequently resulted in the suppression of tumor angiogenesis.

MMP activity is regulated by its gene expression and proenzyme activation. Numerous reports have demonstrated that MMP-2 and MMP-9 expression are critically mediated by MAPK members and the PI3K/Akt pathway [34], [36], [37]. MAPK and PI3K/Akt pathways also play an important role in tumor development and progression [30], [34], [54]. Therefore, we examined the effect of diosgenin on the activities of MAPK and PI3K/Akt signaling pathways. The results demonstrated that treatment with diosgenin inhibited ERK, JNK, PI3K and Akt phosphorylation significantly, suggesting that the signaling pathways mediated by ERK, JNK and PI3K/Akt were suppressed by diosgenin. In addition, we showed that inhibitors of ERK, JNK and PI3K suppressed cell invasion and MMP-2/9 mRNA expression of PC-3 cells significantly. Therefore, we suggested that diosgenin inhibited invasion of PC-3 cells might partly through suppressing ERK, JNK and PI3K pathways. ERK, JNK and PI3K/Akt pathways may be the potential targets for suppressing prostate cancer metastasis.

Recent studies have demonstrated that inhibition of NF-κB activity could suppress metastasis [31], [33]. In the present study, we demonstrated that diosgenin elevated IκBα protein level in the cytoplasm and abolished NF-κB protein level in the nucleus, suggesting that diosgenin suppressed the activity of NF-κB. Hence, the inhibitory effect of diosgenin on NF-κB may be involved in the anti-metastatic mechanisms of diosgenin.

While we suggested diosgenin could inhibit PC-3 cell migration and invasion through suppressing ERK, JNK, PI3K as well as NF-κB pathways, whether the effect of diosgenin on expression of MMP-2/9/7, TIMP-2, EMMPRIN and VEGF were mediated by these pathways was not confirmed. Therefore, using RNAi technique that specifically knockdown the expression of ERK, JNK and PI3K to investigating the role of these signaling pathways on expression of MMP-2/9/7, TIMP-2, EMMPRIN and VEGF as well as cell invasion will be carried out in future.

In the present study, we demonstrated the inhibitory effect of diosgenin on proliferation, migration and invasion of human prostate cancer PC-3 cell. We also observed that diosgenin suppressed proliferation and invasion of human lung carcinoma A549 cell (unpublished data). Thus, diosgenin may have potential for anti-metastatic therapy. Diosgenin is considered safe since it does not manifest systemic toxicity, genotoxicity and estrogenic activity [55]. Diosgenin is thought to be neither synthesized nor metabolically converted into steroid by-products in the mammalian body [1]. However, diosgenin is one of the aglycone of steroidal saponins which may cause hemolysis after intravenous administration and be quickly hydrolyzed after oral administration [56]. This elicits the question that within a clinical perspective the use of diosgenin could be compromised. Therefore, investigating the therapeutic potential and pharmacodynamic property of diosgenin in vivo is imperative. The animal study for investigating the effect of diosgenin on prostate cancer metastasis will be implemented in future.

In conclusion, we attributed the decrease in expression and activity of MMP-2/9 by diosgenin to the inhibition of the ERK, JNK and PI3K/Akt signaling pathways as well as NF-κB activity, and such suppressive effect might contribute to the inhibition of migration and invasion in human prostate cell PC-3 by diosgenin. These findings reveal a new therapeutic potential for diosgenin on anti-metastatic therapy.
